# At the Nexus Between Epigenetics and Senescence: The Effects of Senolytic (BI01) Administration on DNA Methylation Clock Age and the Methylome in Aged and Regenerated Skeletal Muscle

**DOI:** 10.1111/acel.70068

**Published:** 2025-04-21

**Authors:** Toby L. Chambers, Jaden Wells, Pieter Jan Koopmans, Francielly Morena, Zain B. Malik, Nicholas P. Greene, Antonio Filareto, Michael Franti, Patrizia Sini, Harald Weinstabl, Robert T. Brooke, Milda Milčiūtė, Juozas Gordevičius, Steve Horvath, Yuan Wen, Cory M. Dungan, Kevin A. Murach

**Affiliations:** ^1^ Exercise Science Research Center, Molecular Muscle Mass Regulation Laboratory, Department of Health, Human Performance, and Recreation University of Arkansas Fayetteville Arkansas USA; ^2^ Department of Health, Human Performance, and Recreation Baylor University Waco Texas USA; ^3^ University of Arkansas Cell and Molecular Biology Graduate Program Fayetteville Arkansas USA; ^4^ Boehringer Ridgefield Connecticut USA; ^5^ Boehringer Vienna Austria; ^6^ Epigenetic Clock Development Foundation Los Angeles California USA; ^7^ Department of Human Genetics University of California Los Angeles Los Angeles California USA; ^8^ Altos Labs San Diego California USA; ^9^ University of Kentucky Center for Muscle Biology Lexington Kentucky USA; ^10^ Department of Physiology University of Kentucky Lexington Kentucky USA; ^11^ Division of Biomedical Informatics, Department of Internal Medicine University of Kentucky Lexington Kentucky USA

**Keywords:** aging, DNAmAGE, extracellular matrix, methylation clock, omics integration

## Abstract

Senescent cells emerge with aging and injury. The contribution of senescent cells to DNA methylation age (DNAmAGE) in vivo is uncertain. Furthermore, stem cell therapy can mediate “rejuvenation”, but how tissue regeneration controlled by resident stem cells affects whole tissue DNAmAGE is unclear. We assessed DNAmAGE with or without senolytics (BI01) in aged male mice (24–25 months) 35 days following muscle healing (BaCl_2_‐induced regeneration versus non‐injured). Young injured mice (5–6 months) without senolytics were comparators. DNAmAGE was decelerated by up to 68% after injury in aged muscle. DNAmAGE was modestly but further significantly decelerated by injury recovery with senolytics. ~1/4 of measured CpGs were altered by injury then recovery regardless of senolytics in aged muscle. Specific methylation changes caused by senolytics included differential regulation of *Col*, *Hdac*, *Hox*, and *Wnt* genes, which likely contributed to improved regeneration. Altered extracellular matrix remodeling using histological analysis aligned with the methylomic findings with senolytics. Without senolytics, regeneration had a contrasting effect in young mice and tended not to influence or modestly accelerate DNAmAGE. Comparing young to old injury recovery without senolytics using methylome‐transcriptome integration, we found a more coordinated molecular profile in young and differential regulation of genes implicated in muscle stem cell performance: *Axin2*, *Egr1*, *Fzd4*, *Meg3*, and *Spry1*. Muscle injury and senescent cells affect DNAmAGE and aging influences the transcriptomic‐methylomic landscape after resident stem cell‐driven tissue reformation. Our data have implications for understanding muscle plasticity with aging and developing therapies aimed at collagen remodeling and senescence.

## Graphical abstract

Injury then recovery markedly rewires the DNA methylome in aged skeletal muscle.The addition of senolytics during muscle regeneration decelerates DNAmAGE more than regeneration alone as well as targets collagen remodeling and stem cell function‐related genes. Regeneration in young adult mice has a less pronounced effect on the methylome‐transcriptome landscape than in aged skeletal muscle, but still elicits a distinct molecular profile versus aged skeletal muscle after injury.

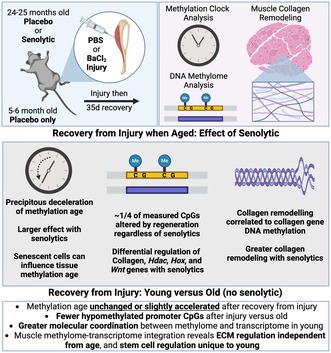

**Abbreviations**
*Asb*
: ankyrin repeat and SOCS box protein
BH: Benjamini‐Hochberg
*Col*
: collagen
DM: differentially methylated
DNAmAGE: DNA methylation age
ECM: extracellular matrix
FAPs: fibroadipogenic progenitors
*Fgfrl1*
: fibroblast growth factor receptor‐like 1
*Fzd5*
: frizzled class receptor 5
GSEA: Gene Set Enrichment Analysis
*Hdac*
: histone deacetylase
*Hox*
: Homebox
*Mapk*
: Mitogen‐activated protein kinases
OS: old senolytic
OV: old vehicle
PBS: phosphate buffered saline
SeSAMe: Sensible Step‐wise Analysis of Methylation Data
*Smad4*
: SMAD family member 4
TA: tibialis anterior
TSS: transcription start site
*Wnt*
: wingless‐type MMTV integration site family
YV: young vehicle

## 1 Background

DNA methylation clocks accurately predict the chronological age of an organism (Hannum et al. 2013; Horvath 2013; Horvath and Raj 2018). Building on this discovery, a disparity between chronological age and DNA methylation age (DNAmAGE) can be indicative of biological aging and have consequences for predicting healthspan and lifespan (Bell et al. 2019; Bernabeu et al. 2023; Moqri et al. 2024). DNAmAGE is therefore used as a biomarker to infer accelerated or decelerated aging resulting from different conditions across tissues and organisms (Unnikrishnan et al. 2019). There is utility in determining DNA methylation age as it is linked to cellular and tissue dysfunction or restoration (Olova et al. 2019; Poganik et al. 2023; Zhang, Lee, et al. 2023), and it may be causative of deleterious aging phenotypes (Ying et al. 2024).

The emergence of replication‐incompetent senescent cells is a “hallmark” or “pillar” of aging across tissues and organisms that may lead to local and systemic dysfunction (Kennedy et al. 2014; López‐Otín et al. 2013, 2023). These hallmarks, which include aging‐mediated epigenetic alterations, can be distinct but also overlapping. Replicative senescence is associated with an increase in DNAmAGE in cultured cells (Lowe et al. 2016), and DNAmAGE can track with cell passage number in vitro (Koch and Wagner 2013; Liu et al. 2020; Sturm et al. 2019). Since tissues are comprised of heterogeneous cell types, it is conceivable that an accumulation of senescent cells may drive DNAmAGE analyses at the whole tissue level (Liu et al. 2020). To this point, differentiation stage, replicative history, and cell type composition can affect DNAmAGE in muscle cells and other tissues (Gorelov et al. 2024). Other forms of inducing cellular senescence such as radiation or oncogene expression appear to be disconnected from an increase in DNAmAGE (Kabacik et al. 2018, 2022; Lu et al. 2018); this aligns with Horvath's hypothesis in his original work on the pan tissue methylation aging clock (Horvath 2013). Whether removing senescent cells in vivo influences DNAmAGE is not clear (Wagner 2019). This question is complicated by the fact that senescent cells may be detrimental or beneficial depending on the tissue and context (Baker et al. 2023; Dungan et al. 2023; Saito et al. 2020; Young et al. 2022).

Senescent cells and muscle fiber nuclei (i.e., myonuclei of syncytial muscle fibers) with senescent features may manifest with aging in skeletal muscle (Dungan et al. 2023; Moiseeva et al. 2023; Perez et al. 2022; Zhang, Habiballa, et al. 2022). Cells with a senescence signature also appear after severe injury in skeletal muscle regardless of age but persist in aged tissue after recovery (Dungan et al. 2021, 2023; Nolt et al. 2024). We reported that treatment with senolytic compounds improved regeneration after severe muscle injury in aged skeletal muscle (Dungan et al. 2021). We also showed that senolytics rescue the blunted hypertrophic response to skeletal muscle loading in aged mice (Dungan et al. 2022; Nolt et al. 2024). In the current investigation, we used skeletal muscle tissue from mice treated with a novel and highly effective senolytic compound (BI01) (Nolt et al. 2024) to determine whether senolytic treatment affects DNAmAGE in skeletal muscle after injury. We previously reported on how this compound specifically targets p53‐MDM2, reduces the abundance of senescent mononuclear cells, and improves muscle regeneration (Nolt et al. 2024). Additionally, we establish whether regeneration of skeletal muscle by resident stem cells—which are the same chronological age as the tissue itself—affects tissue DNAmAGE in aged versus young muscle in the absence of senolytics (Horvath 2013). Finally, we provide fundamental information on the methylation landscape in young and aged skeletal muscle after regeneration and how this relates to muscle gene expression using an omics data integration technique called BETA that we have validated in muscle (Ismaeel et al. 2024). Our findings uncover the relationship between DNAmAGE and senescent cell accumulation in vivo, as well as illustrate how the methylome and transcriptome is rewired following muscle regeneration.

## 2 Methods

### 2.1 Animals

**FIGURE 1:**
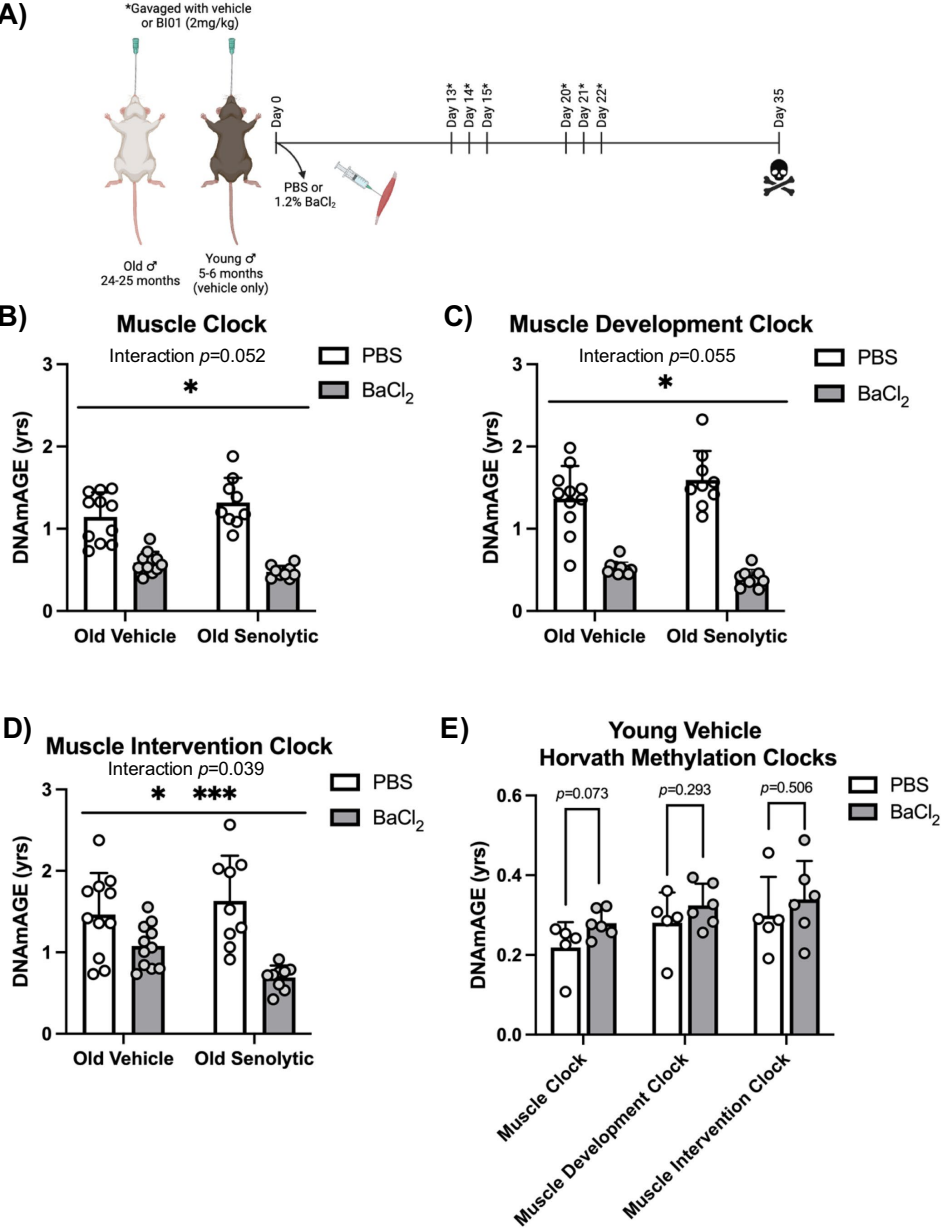
Study design and Horvath DNAmAGE clocks. (A) Study design schematic that shows the timing of BaCl_2_ injury (Day 0), drug administration as denoted with the asterisk (*), and euthanasia (skull & crossbones icon). DNA methylation age (DNAmAGE) derived from clocks developed by the Horvath laboratory applied to old mice 35 days after muscle injury with and without senolytic treatment for (B) muscle, (C) muscle development, and (D) Muscle Intervention clocks. Shown also are DNAmAGE in young mice without senolytics 35 days after muscle injury for (E) muscle, muscle development, and muscle intervention clocks. *main effect for injury (*p* < 0.05); ***interaction effect (*p* < 0.05).

Old (24–25 month; *n* = 20) and young (5–6 month; *n* = 5–6) male C57BL/6J mice (Jackson Labs, Bar Harbor, ME, USA) were used to determine the effects of BaCl_2_ and/or senolytics on skeletal muscle DNA methylation status. The tissue for this study is that which was used in our previous study, where all experimental details can be found (Nolt et al. 2024). [Correction added on 13th May 2025 after first online publication: The year of publication has been corrected to “2024.” All in‐text citations have been updated accordingly throughout the text.] All animal procedures were approved by the IACUC of the University of Kentucky. Mice were housed in a temperature and humidity‐controlled room, maintained on a 14:10‐h light–dark cycle with food and water provided *ad libitum*. Mice were euthanized following a 6 h fasting period via exsanguination under isoflurane anesthesia, followed by cervical dislocation. Following the removal of the tibialis anterior (TA), the muscles were weighed, and a piece of the TA was cut longitudinally, flash frozen in liquid nitrogen and stored at −80°C until total RNA and DNA isolation (one half of TA), or histology (other half of TA). In the current investigation, we used Horvath 320k mammalian methylation assays to explore differences in methylated CpG sites 35 days following degenerative muscle injury (i.e., intramuscular BaCl_2_ injection) versus control (PBS injected muscle). Old mice were treated with a senolytic agent (OS, *n* = 9) or vehicle (OV, *n* = 11) during the 35‐day post‐induced injury period. Young mice (YV) were only treated with vehicle (no senolytics) and BaCl_2_ (*n* = 6) or PBS (*n* = 5) injections in contralateral TA muscles. The study design schematic is presented in Figure 1A.

### 2.2 BaCl_2_ Injections

Barium chloride (BaCl_2_) causes a chemical injury that mimics muscle damage by inhibiting K^+^ channels, leading to depolarization, causing Ca^++^ overload, proteolysis and membrane disruption (Hardy et al. 2016). This model permits study of aspects of the natural repair processes and the impacts of various treatments, including senolytics, on muscle regeneration. Injections of BaCl_2_ were performed as previously described by our group (Dungan et al. 2021; Nolt et al. 2024). Briefly, a 1.2% BaCl_2_ (342920, Sigma‐Aldrich, St. Louis, MO) solution was injected into the left TA at five locations equally spaced along the length of the muscle with 10 μL of 1.2% BaCl_2_ at each location. The right TA muscle acted as a control, which was injected with phosphate buffered saline (PBS) in a similar manner. In the present investigation, mice were euthanized 35 days following BaCl_2_ injections. The mice were injected with PBS or BaCl_2_ in contralateral TA muscles on day 0 of the study protocol.

### 2.3 Administration of BI01 Senolytic Agent

The novel senolytic agent BI01 was developed by Boehringer Ingelheim Pharmaceuticals Inc. (Ingelheim, Germany) with > 99.5% purity. BI01 was dissolved in vehicle (0.5% 2‐hydroxyethyl cellulose; 434981, Sigma‐Aldrich) and administered to the mice via oral gavage using a polypropylene feeding tube (FTP‐20‐20, Instech, Plymouth Meeting, PA) at a concentration of 2 mg/kg. To ensure that we did not exceed the capacity of the average murine stomach, BI01 was dissolved in vehicle at 0.06 mg per 100 μL (enough BI01 for a 30 g mouse). Mice were weighed prior to each gavage, with gavage volumes ranging from 80 to 150 μL depending on the weight of the mouse. Administration of the senolytic compound occurred on days 13, 14, 15, 20, 21, and 22 after injury. The pharmacokinetics of BI01, dosing strategy, and validation of p53‐MDM2 targeting have been previously reported by us, and BI01 significantly lowered senescent cell burden (SA β‐Gal and p21+ cells) in response to regeneration and improved muscle mass, function, and cellular outcomes (Nolt et al. 2024).

### 2.4 DNA Methylation Profiling for DNAmAGE

DNA was extracted from flash frozen TA skeletal muscle tissue using a QIAamp DNA Micro kit (catalog. No. 56304; Qiagen, Hilden, Germany) in accordance with manufacturer guidelines and eluted using ultrapure, nuclease‐free MilliQ water (Millipore Corp., Burlington, MA, USA). DNA purity and concentration were assessed via a 260/280 absorption ratio. A subset of samples was further quality‐checked using a Genomic DNA ScreenTape on the Agilent Tapestation Controller (Agilent Technologies, Santa Clara, California) to ensure non‐degraded DNA. Average DIN was 8.6 ± 0.9, and DNA fragment size was generally > 50,000 bp. The isolated DNA was then shipped to Clock Foundation and underwent bisulfite conversion using the Zymo EZ DNA methylation kit (Zymo, Irvine, CA, USA). A minimum of 150 ng of total bisulfite‐converted DNA was processed using the Horvath 320k methylation assay (Clock Foundation, Los Angeles, CA, USA). The HorvathMammalMethylChip320 custom mammalian array was used, which combines a prior custom mammalian array (HorvathMammalMethylChip40) and the 285k Illumina Mouse Methylation BeadChip (Arneson et al. 2022). Raw data were normalized using the Sensible Step‐wise Analysis of Methylation data (SeSAMe) R package (Zhou et al. 2018), resulting in a methylation estimate (beta value) corresponding to each array probe for every individual in the dataset and a detection *p* value corresponding to the confidence in the normalized beta value. Beta values from SeSAMe are derived from the ratio of the fluorescence intensity of a methylated probe for a specific CpG to the total overall probe intensity (the sum of the signal from both the methylated and unmethylated probes plus a constant) (Du et al. 2010). Beta values range from zero to one, with a value of zero indicating that no copies of the gene were methylated. In order to transform a 320k array to 40k for predictive analysis, we employ probes that align between both arrays with a correlation exceeding 0.8. For any missing probes, we assign gold median values determined through calculations using a substantial mouse reference dataset. We used published epigenetic clocks for mice that were trained on independent data as previously described (Mozhui et al. 2022). The R software code underlying our mouse methylation clocks is implemented in the R package MammalMethylClock (Zoller and Horvath 2024). The methylation aging clocks used in the present study to determine DNAmAGE were the muscle, muscle development, and muscle intervention clocks. These specific Horvath methylation clocks are based on CpGs from muscle tissue (muscle clock), CpGs that change during the initial 6 weeks of mouse development (muscle development), and CpGs that change after an anti‐aging intervention (muscle intervention).

### 2.5 Global Methylation Analysis

Normalized beta values were used to fit the linear regression model using the R package *limma* v3.50.3 (Ritchie et al. 2015). To remove the effect of known confounders, the multivariate linear model used sample group and sentrixID as covariates. Adjustment for unknown confounders was performed using the R package RUVSeq v1.28.0, and one RUVg vector was used to account for these confounders (Risso et al. 2014). The defined model was used to identify differentially methylated cytosines in several different contrasts. The model was fitted using the least‐squares method by running the lmFit function. Empirical Bayes statistics were estimated using the eBayes function. The Benjamini‐Hochberg (BH) method was used to correct for multiple testing. Probes with a *q*‐value < 0.05 were deemed significant.

### 2.6 Gene Set Enrichment Analysis (GSEA)

Functional analyses were conducted using GSEA (v4.1.0) software (Mootha et al. 2003; Subramanian et al. 2005). Genes were ranked based on the sign of their differential methylation fold change and negative log10‐transformed *p* value. Known mouse gene sets were obtained from the Bader lab website on May 1st, 2023. The GMT file was filtered using the GSEA software to include only pathways with the gene count between 5 and 500. Pathway enrichment *p* values were estimated through 1000 permutations. Subsequently, all significantly enriched pathways were clustered, and their interaction networks were visualized using aPEAR package (Kerseviciute and Gordevicius 2023). Each cluster was named after the most influential pathway within that cluster, determined using the PageRank algorithm.

### 2.7 RNA Isolation and RNA‐Seq Analysis

Full details on the RNA isolation and RNA‐seq analysis can be found in Nolt et al. (2024). Briefly, total RNA was isolated using the Qiagen miRNeasy Mini Kit (Cat. #217004, Qiagen, Germantown, MD) per manufacturer guidelines. The isolated RNA was sent to Novogene Co. (Beijing, China) for library preparation and mRNA sequencing. Bioinformatic analysis [*Partek Flow* software, v10.0 (St. Louis, MO)] and pre‐alignment quality control were completed. Alignment of sequencing reads to the mouse genome (GRCm39) using splice‐aware program STAR (v2.7.8a). Differential gene expression was analyzed using DESeq2 (v3.5).

### 2.8 BETA Integration of Methylomics and RNA‐Seq Data

We have presented the integration of epigenetic and RNA‐seq data using BETA in a previous publication from Ismaeel et al. (2024). BETA was developed as a software that provides an integrated analysis of transcription factor binding to genomic DNA and transcript abundance using chromatin immunoprecipitation sequencing (ChIP‐seq) and transcriptomics (RNA‐seq) datasets (Wang et al. 2013). The underlying algorithm of BETA considers the distance of regulatory elements relative to the transcriptional start site (TSS) by modeling the effect of regulation using a natural log function, termed the regulatory potential, as described previously in the work by Tang et al. (2011). In this study, we adapted BETA to integrate CpG methylomics with RNA‐seq data sets after induced TA muscle injury. CpGs (whether hypo‐ or hyper‐methylated) were converted to “methylation peaks” similar to transcription factor binding peaks. Only genes differentially expressed with adjusted *p* values < 0.05 from RNA‐seq analysis were included as input for gene expression. Further details and specifics on the technical aspects of the BETA analysis used for this investigation can be found in Ismaeel et al. (2024). Differentially expressed genes (adj. *p* value < 0.05) using gene symbols were uploaded into ShinyGO (http://bioinformatics.sdstate.edu/go/) (Ge et al. 2020) using our entire gene list as a background input and overrepresentation analysis for GO molecular function enrichment was performed using an FDR cutoff of 0.05. Figures were extracted from ShinyGO. All other figures were generated using GraphPad Prism v.10.2.2 for Mac OS (GraphPad Software, La Jolla, CA).

### 2.9 Histological Analysis of Skeletal Muscle Collagen Remodeling

Skeletal muscle samples were frozen in OCT and sectioned using an HM525NX cryostat (Thermo Fisher) at −24°C, and 20 μm sections were stored at −80°C until the time of analysis. To assess dynamic collagen remodeling, muscle sections were stained using a collagen hybridizing peptide (R‐CHP) conjugate (3Helix, Salt Lake City, UT). Muscle sections were allowed to air dry for 60 min before fixation in cold acetone (−20°C) for 10 min, rinsed in PBS (3 × 3 min), and blocked in 2.5% normal horse serum for 60 min at room temperature. R‐CHP‐Cy3 conjugate (3Helix) was diluted with PBS to a working solution (20 μM) and placed on a heating block at 80°C for 5 min to denature 3Helix trimers, then quickly cooled on wet ice for 2 min. Immediately after cooling, R‐CHP‐Cy3 was added to the sections, then incubated for 60 min at RT and then overnight at 4°C. The next day, slides were rinsed with PBS (3 × 3 min) and then mounted with 50/50 glycerol and PBS before imaging. Images were captured at 20× total magnification at room temperature with a ZEISS upright microscope (Axio Imager M2, Oberkochen, Germany). For each muscle sample, 2–3 images were processed and analyzed for R‐CHP area. Image analysis was performed by the same investigator who was blinded to sample condition throughout the analyses. Quantification of R‐CHP area was performed using ImageJ software (National Institutes of Health, Bethesda, MD) by splitting the images into RGB channels and converting to grayscale. Using the threshold function, binary black and white images were generated for assessment of R‐CHP total percent area per image and normalized per muscle fiber.

### 2.10 Statistical Analyses

A two‐way ANOVA was used with significance set at *p* < 0.05 to determine interaction effects for DNAmAGE between OS and OV drug treatments (senolytics vs. vehicle) 35 days after BaCl_2_ and PBS injections. Two‐tailed unpaired t‐tests with significance set at *p* < 0.05 were used to determine differences 35 days after BaCl_2_ and PBS injections in YV. A two‐way ANOVA with Tukey's post hoc analysis with significance set at *p* < 0.05 was used to determine interaction effects for ECM collagen content (total & unfolded) between OV and OS groups after muscle regeneration with BaCl_2_ and PBS injections.

## 3 Results

### 3.1 Epigenetic Age Was Decelerated in Regenerated Skeletal Muscle of Aged Mice and Was Modestly but Significantly Affected by Senolytics

We previously used murine muscle‐specific DNAmAGE clocks to measure DNAmAGE deceleration after late‐life exercise training (Jones III et al. 2023; Murach, Dungan, et al. 2022). Here, we used clocks to compare the DNAmAGE of skeletal muscle from aged (24–25 month males) senolytic (OS, treated with BI01) and untreated (OV, treated with vehicle) mice 35 days into recovery following unilateral BaCl_2_ regenerative injury; the contralateral leg was PBS‐injected as a control (Nolt et al. 2024). In addition to muscle‐specific clocks, we present the results from pan‐tissue DNAmAGE clocks in Table S1.

There was a main effect for regeneration (BaCl_2_ vs. PBS) according to the muscle clock (*p* = 0.00000000001, Figure 1B), muscle developmental clock (*p* = 0.00000000000008, Figure 1C), and muscle intervention clock (*p* = 0.00001, Figure 1D) in aged mice. DNAmAGE was 56% decelerated (~36 weeks) for the muscle clock (PBS = 63.7 ± 15.9 weeks; BaCl_2_ = 28.0 ± 6.3 weeks), 68% decelerated (~52 weeks) for the muscle developmental clock (PBS = 76.6 ± 20.1 weeks; BaCl_2_ = 24.1 ± 5.6 weeks), and 41% decelerated (~33 weeks) for the muscle intervention clock (PBS = 80.3 ± 27.3 weeks; BaCl_2_ = 47.1 ± 15.3 weeks). A *p* = 0.05 interaction (senolytic treatment × BaCl_2_) was observed for the muscle clock (*p* = 0.052) and the muscle developmental clock (*p* = 0.055), and a significant interaction (*p* < 0.05) was observed for the muscle intervention clock (*p* = 0.039). The interactions were partially driven by a modest acceleration in DNAmAGE in the PBS‐treated OS muscles and deceleration of DNAmAGE with senolytics after regeneration relative to PBS. Overall, senolytic treatment on its own in the absence of regeneration did not affect DNAmAGE according to the muscle clock (*p* = 0.67), muscle developmental clock (*p* = 0.54), or muscle intervention clock (*p =* 0.40) (i.e., no main effect for senolytic was observed).

We measured DNAmAGE 35 days following injury in young vehicle‐treated mice (no senolytic treatment, Table S1). No significance was observed, but there was a trend toward accelerated DNAmAGE after regeneration in young mouse muscle that was observed for the muscle clock (*p* = 0.07), with no difference in muscle development (*p* = 0.29) or muscle intervention clocks (*p* = 0.43) (Figure 1E).

### 3.2 Widespread DNA Methylome Remodeling After Recovery From Injury in Muscle of Aged Mice in the Absence and Presence of Senolytics

**FIGURE 2:**
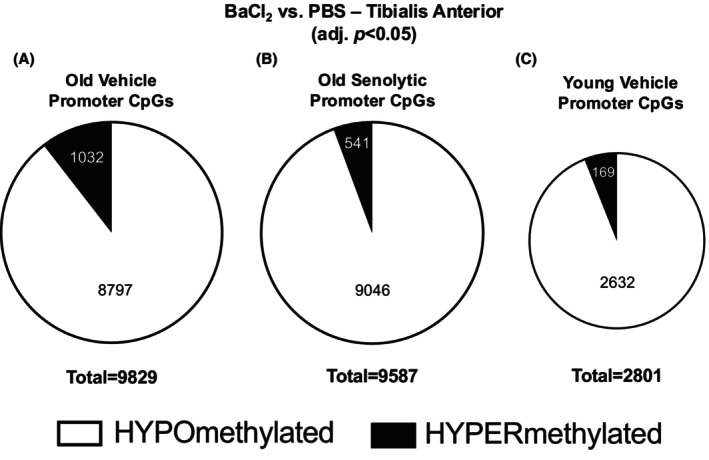
Promoter CpG methylation after muscle regeneration. Total promoter CpG sites differentially methylated after muscle regeneration in (A) old vehicle, (B) old senolytic, and (C) young vehicle.

Little is known about global epigenetic reprogramming that may occur after regeneration in skeletal muscle or the effects of senolytics on the DNA methylome. We therefore sought to define the DNA methylome after regeneration in the absence and presence of senolytics in old mice. In regenerated OV, of ~320,000 CpGs measured, there were 86,759 differentially methylated (DM) CpGs compared to uninjured OV PBS (adj. *p* < 0.05) (Table S2). Of the DM CpGs in OV, 8797 and 1032 were hypo‐ and hypermethylated, respectively, in promoter regions compared to OV PBS (adj. *p* < 0.05, Figure 2). Regeneration in OS resulted in 85,681 DM CpGs relative to PBS (adj. *p* < 0.05) (Table S2). Of the DM CpGs in OS, 9046 and 541 were hypo‐ and hypermethylated CpGs, respectively, in promoter regions compared to OS PBS (adj. *p* < 0.05, Figure 2). In YV, regeneration resulted in 24,393 DM CpGs compared to PBS (adj. *p* < 0.05) (Table S2). Of those DM CpGs in YV, 2632 and 169 were hypo‐ and hypermethylated CpGs, respectively, in promoter regions compared to YV PBS (adj. *p* < 0.05, Figure 2).

Overall, ~26% of all CpGs measured were altered 35 days after degenerative injury in old mice regardless of senolytic administration. Comparatively fewer, but a nevertheless noteworthy number of CpGs were altered by regeneration in young mice (7%, data not shown). Even after muscle mass is fully recovered 35 days following injury, the DNA methylome is markedly reprogrammed in the muscle of old mice, with a more modest but still striking effect in young mice that is consistent with the magnitude of the DNAmAGE results.

### 3.3 DNA Hypomethylation of CpGs in Promoter Regions Relates to Collagens and Tissue Development in Skeletal Muscle of Aged Mice, With Unique Epigenetic Effects of Senolytics on *Hdac*, *Hox*, and *Wnt* Genes

**FIGURE 3:**
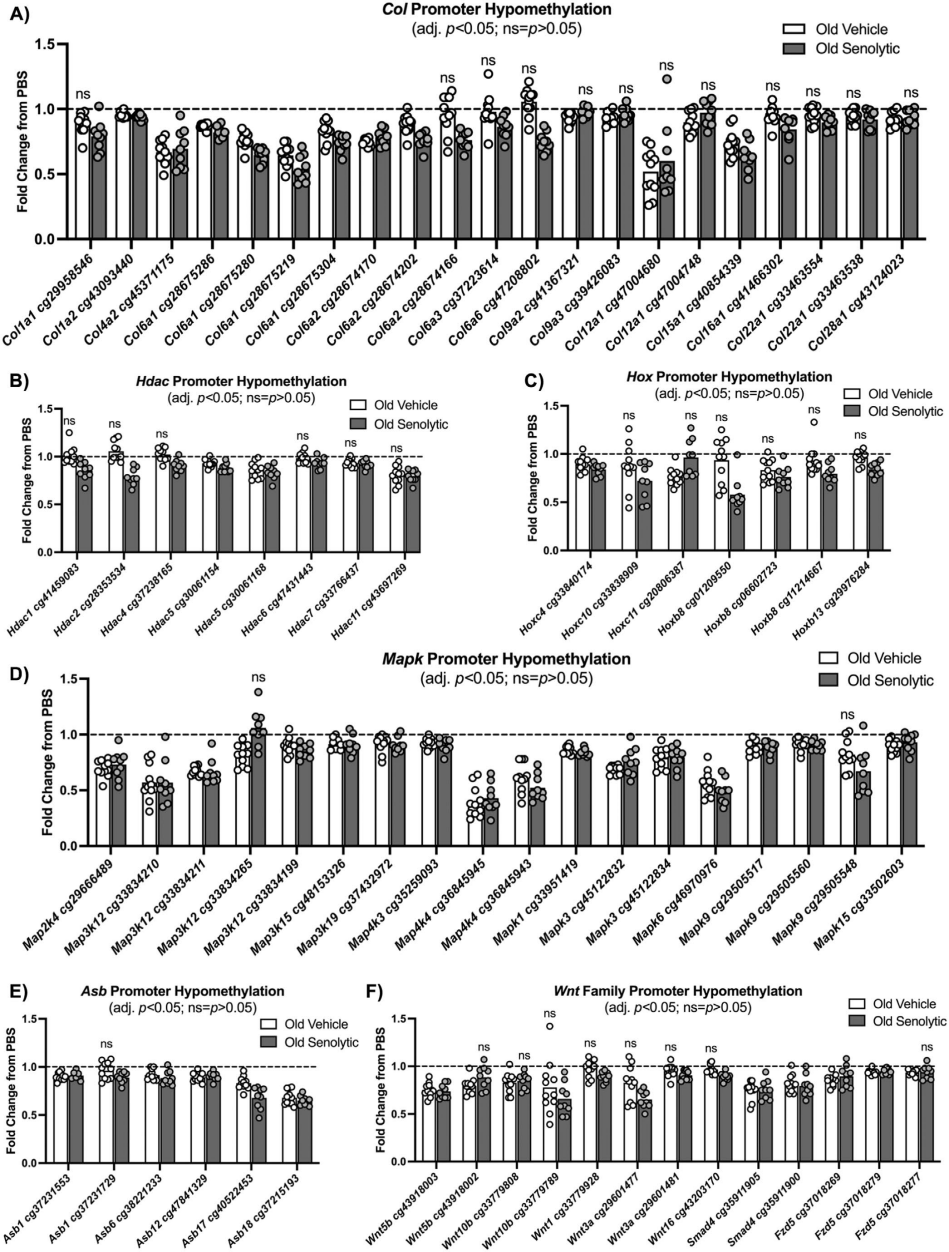
Methylation of conserved promoter CpGs is modified during muscle regeneration in old skeletal muscle. Promoter hypomethylation for old vehicle and senolytic aged skeletal muscle for key gene families: *Col, Hdac, Hox, Mapk, Asb* and *Wnt* signaling (A–F). All promoter CpGs have an adj. *p <* 0.05 unless marked with “ns”, and individual fold change data points presented for old vehicle (*n* = 11) and old senolytic (*n* = 9) are relative to their respective control (PBS) contralateral limb, represented as the line at 1.0. Points/bars above the line mean hypermethylation relative to control, whereas below the line represent hypomethylation. ns, not significant.

Following 35 days of recovery after injury, 24 collagen (*Col*) gene promoter CpGs shared hypomethylation patterns in OV and OS; these included *Col1a2, Col4a2, Col6a1, Col6a2*, and *Col15a1*. In both OV and OS, *Col6a1* and *Col6a2* had multiple promoter CpGs that were hypomethylated following regeneration (Figure 3A). The aforementioned collagens are associated with skeletal muscle integrity and basement membrane maintenance (Eklund et al. 2001; Huang et al. 2024; Kwong et al. 2023; Muona et al. 2002). Promoter hypomethylation for *Col9a2, Col9a3, Col12a1*, and *Col28a1* was only present in OV after regeneration, while *Col1a1, Col6a3, Col6a6, Col16a1*, and *Col22a1* promoter hypomethylation only occurred in OS (adj. *p <* 0.05; Table S3). Since *Col1a1* is the most abundant collagen in skeletal muscle and is most affected during times of muscle stress (Brightwell et al. 2022), our findings therefore point to potentially enhanced collagen remodeling during regeneration in the presence of senolytics. High levels of collagen expression may be viewed as pathologic, but elevated collagen expression is also characteristic of healthy skeletal muscle in the context of exercise and muscle hypertrophy (Brightwell et al. 2022).

Members of the histone deacetylase (*Hdac*) gene family had hypomethylated CpGs in promoter regions in regenerated muscle of OV and OS. In OV, *Hdac5 and Hdac11* had hypomethylated CpGs in promoter regions following regeneration (adj. *p* < 0.05). In OS, *Hdac1, Hdac2, Hdac4, Hdac5, Hdac6, Hdac7*, and *Hdac11* had hypomethylated promoter CpGs following regeneration (adj. *p* < 0.05; Figure 3B). In both OV and OS, *Hdac5* had multiple promoter CpGs that were hypomethylated after regeneration. The *Hdac* family of genes plays vital roles in skeletal muscle remodeling, myotube differentiation, and metabolism (Bassel‐Duby and Olson 2006; Egan and Sharples 2023; Fisher et al. 2017; Gorski et al. 2023; Martin et al. 2023; McGee et al. 2014; Núñez‐Álvarez et al. 2021; Simmons et al. 2011; von Walden et al. 2020). Given the central role of HDACs in satellite cell behavior, methylation changes to these genes after muscle regeneration could represent an epigenetic memory of satellite cell identity within muscle fibers after fusion (Falick Michaeli et al. 2022; Morroni et al. 2023). We recently provided initial evidence for an epigenetic “memory” in myonuclei derived from satellite cells after exercise training (Murach, Dungan, et al. 2022). Persistent *Hdac4* promoter CpG hypomethylation—in the myonuclei and/or in satellite cells—with senolytics could be related to this HDAC's known role in regulating satellite cell function and facilitating regeneration (Choi et al. 2014; Marroncelli et al. 2018; Renzini et al. 2018). Methylation differences in *Hdac1* and *Hdac2* specifically in OS could also be related to changes within myonuclei that contribute to muscle fiber growth, as has been reported with mechanical overload (von Walden et al. 2020). Exaggerated hypomethylation of *Hdac* genes in the presence of senolytics may therefore have contributed to improved regeneration in these mice that we previously reported (Nolt et al. 2024). It is also known that aberrant HDAC activity in muscle fibroadipogenic progenitors (FAPs) affects regenerative ability and ECM deposition (Consalvi et al. 2022; Mozzetta et al. 2013). Thus, this unique methylation signature of *Hdac* genes in the presence of senolytics may also be attributable to the FAP methylome in muscle tissue after regeneration.

Several transcriptional regulators had hypomethylated promoter region CpGs following regeneration in both OV and OS muscle. Homebox C4 (*Hoxc4*) was hypomethylated at the same promoter site in OV and OS. In OV only, *Hoxc11* was hypomethylated at promoter region CpGs. In OS only, *Hoxb8, Hoxc10*, and *Hoxb13* had promoter region CpG hypomethylation after regeneration (adj. *p* < 0.05; Figure 3C). *Hox* genes are the drivers of tissue development (Krumlauf 1994) and are differentially regulated by DNA methylation in young and old skeletal muscle and with exercise (Tsumagari et al. 2013; Turner et al. 2020). As we previously reported, the methylation of *Hox* genes is affected by myonuclear accretion with exercise in murine myonuclei (Murach, Dungan, et al. 2022), highlighting a potential role for these genes in muscle adaptation. *Hox* transcription factors can control myogenic genes (Houghton and Rosenthal 1999; Yamamoto and Kuroiwa 2003; Yoshioka et al. 2021), so persistent hypomethylation of these genes after regeneration could be related to this molecular function. It is also worth noting that activation of certain *Hox* genes, such as *Hoxa9*, can be deleterious to satellite cell function and impair regeneration during aging (Schwoerer et al. 2016)—but we did not observe effects in *Hoxa9*.

Mitogen‐activated protein kinases (*Mapk*) genes had hypomethylated CpGs in both OV and OS skeletal muscle after regeneration (*Map2k4, Map3k12, Map3k15, Map3k19, Map4k3, Map4k4, Mapk1, Mapk3, Mapk6, Mapk9, Mapk15*; Figure 3D) (adj. *p* value < 0.05). Multiple hypomethylated CpG promoter sites were present for *Map3k12, Map4k4, Mapk3*, and *Mapk9* after regeneration (adj. *p* value < 0.05). Activation of MAPK pathways in skeletal muscle may drive metabolic adaptations related to glucose transport and oxidative capacity in response to cellular stress such as exercise (Kramer and Goodyear 2007). Promoter hypomethylation of *Mapk* genes observed here may participate in preventing metabolic dysregulation during regeneration to aid in the restoration of skeletal muscle mass and function (Alway et al. 2023; Cargnello and Roux 2011; Guo et al. 2020).

**FIGURE 4:**
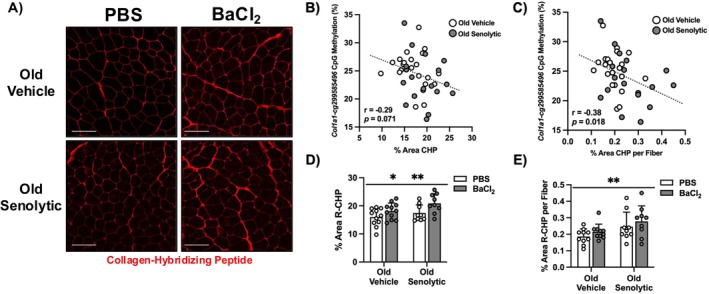
Collagen remodeling and content is enhanced after regeneration following senolytic treatment in skeletal muscle of old mice. (A) Representative histochemical images of R‐CHP stain for OV and OS mice with PBS or BaCl_2_ conditions. Scale bar = 100 μm. (B) Relationship between *Col1a1* CpG methylation (%) to total R‐CHP area (%) across all old mouse samples (*n* = 40) (*r* = −0.29, *p =* 0.071). (C) Relationship between *Col1a1* CpG methylation (%) to total R‐CHP area per muscle fiber (%) across all old mouse samples (*n* = 39) (*r* = −0.38, *p =* 0.018). (D) Quantification of R‐CHP content of tibialis anterior represented as mean percentage of total muscle area ± SD of OV (*n* = 11) and OS (*n* = 9) mice after regeneration. (E) Quantification of R‐CHP area per muscle fiber ± SD of OV (*n* = 10) and OS (*n* = 9). *main effect for injury (*p* < 0.05); **main effect for senolytics (*p* < 0.05).

In both OV and OS, ankyrin repeat and SOCS box protein (*Asb*) *Asb1, Asb6, Asb12, Asb17*, and *Asb18* had similar promoter hypomethylation patterns after regeneration (adj. *p* value < 0.05; Figure 3E). Multiple CpG promoter sites were hypomethylated in *Asb1* after regeneration only in OS (adj. *p* value < 0.05; Figure 4E). The ASB protein family is implicated in skeletal muscle mass regulation, responsiveness to overload, and muscle disease (Ehrlich et al. 2020; Gilbert et al. 2024). ASBs typically function as E3 ubiquitin ligases and ASB6 is involved in autophagy (Gong et al. 2021) and insulin signaling (Wilcox et al. 2004). The roles of other *Asb* genes in skeletal muscle are related to protein synthesis, differentiation, as well as the proliferation and maintenance of the muscle progenitor compartment (Tee and Peppelenbosch 2010). Recently, we reported ASB6 protein upregulation in skeletal muscle after late‐life exercise training in mice (Chambers et al. 2024). Our data collectively reinforce the involvement of *Asb* genes in skeletal muscle remodeling and that these genes are controlled by DNA methylation, but more work is needed on their specific cellular functions in muscle.

Several members of the wingless‐type MMTV integration site family (*Wnt*) genes had hypomethylated CpGs in promoter regions in OV (*Wnt5b* and *Wnt10b*) and in OS (*Wnt1, Wnt3a, Wnt5b, Wnt10b*, and *Wnt16*) (adj. *p* value < 0.05; Figure 3F). Multiple promoter CpGs were hypomethylated for *Wnt5b* in OV, while *Wnt3a* had multiple CpG promoters that were hypomethylated after regeneration in OS (adj. *p* value < 0.05). Among OV and OS, both featured CpG promoter hypomethylation for *Wnt5b* and *Wnt10b* but at differing CpG promoter sites after regeneration. Both groups also had hypomethylation in multiple promoter CpGs of other canonical *Wnt* pathway genes including SMAD family member 4 (*Smad4*) and frizzled class receptor 5 (*Fzd5*) (Figure 3F), the latter of which is a *Wnt* receptor.

Overall, degeneration then regeneration had a powerful effect on reprogramming the skeletal muscle DNA methylome of aged mice with a modest but significant effect of senolytics. On a gene‐by‐gene basis, promoter region epigenetic remodeling of *Col*, *Hdac, Hox*, and *Wnt* genes mediated by senolytic treatment may contribute to enhanced satellite cell function, ECM remodeling, and subsequent enhanced regeneration in the OS group (Moiseeva et al. 2023).

#### 3.3.1 Collagen Remodeling Is Augmented Following Senolytic Treatment and Regeneration, Consistent With the Methylome Data

We recently reported on the methylome regulation of the proteome following late‐life exercise in mice (Chambers et al. 2024). These data established a link between DNA methylation and protein outcomes of aged skeletal muscle in response to stress. Given the apparent epigenetic regulation of collagen genes that differed between OV and OS, we assessed collagen remodeling via R‐CHP staining on muscle cross sections. R‐CHP only binds to unfolded triple‐helical proteins of all collagen types (Abramowitz et al. 2018; Hwang et al. 2017; Li et al. 2013), thereby serving as a readout of collagen remodeling. Representative images for R‐CHP for OV and OS are shown in Figure 4A. First, to establish a link between DNA methylation status and histological measures of collagen remodeling, we correlated promoter methylation levels of the most transcriptionally abundant collagen in muscle (Brightwell et al. 2022)—*Col1a1*—to R‐CHP across all samples. There was a *p* < 0.10 negative correlation for percent area of R‐CHP to *Col1a1* promoter CpG methylation levels (*r* = −0.29, *p* = 0.071) and a significant negative correlation for percent R‐CHP area per fiber (*r* = −0.38, *p* = 0.017) (Figure 4B,C). This relationship suggests that collagen promoter region hypomethylation relates to collagen remodeling in murine muscle tissue, similar to how DNA methylation levels of numerous gene ontologies relate to the global proteome with exercise in aged muscle (Chambers et al. 2024). We then compared collagen remodeling in OV versus OS after recovery from injury. There were main effects for muscle regeneration after injury (*p =* 0.007; total R‐CHP area) and senolytics (*p* = 0.038 and *p* = 0.01; total R‐CHP area and R‐CHP area per fiber, respectively) in aged mice (Figure 4D,E). The main effect of treatment (vehicle vs. senolytics) was driven by elevated total and relative R‐CHP content in both PBS and BaCl_2_ treated OS TA muscle. This finding suggests an overall effect of senolytics on collagen remodeling that dovetails with the epigenetic findings and may be permissive for improved regeneration.

### 3.4 Without Senolytics, Hypomethylation of CpGs in Promoter Regions Is Attenuated in Young Relative to Aged Muscle After Regeneration

**FIGURE 5:**
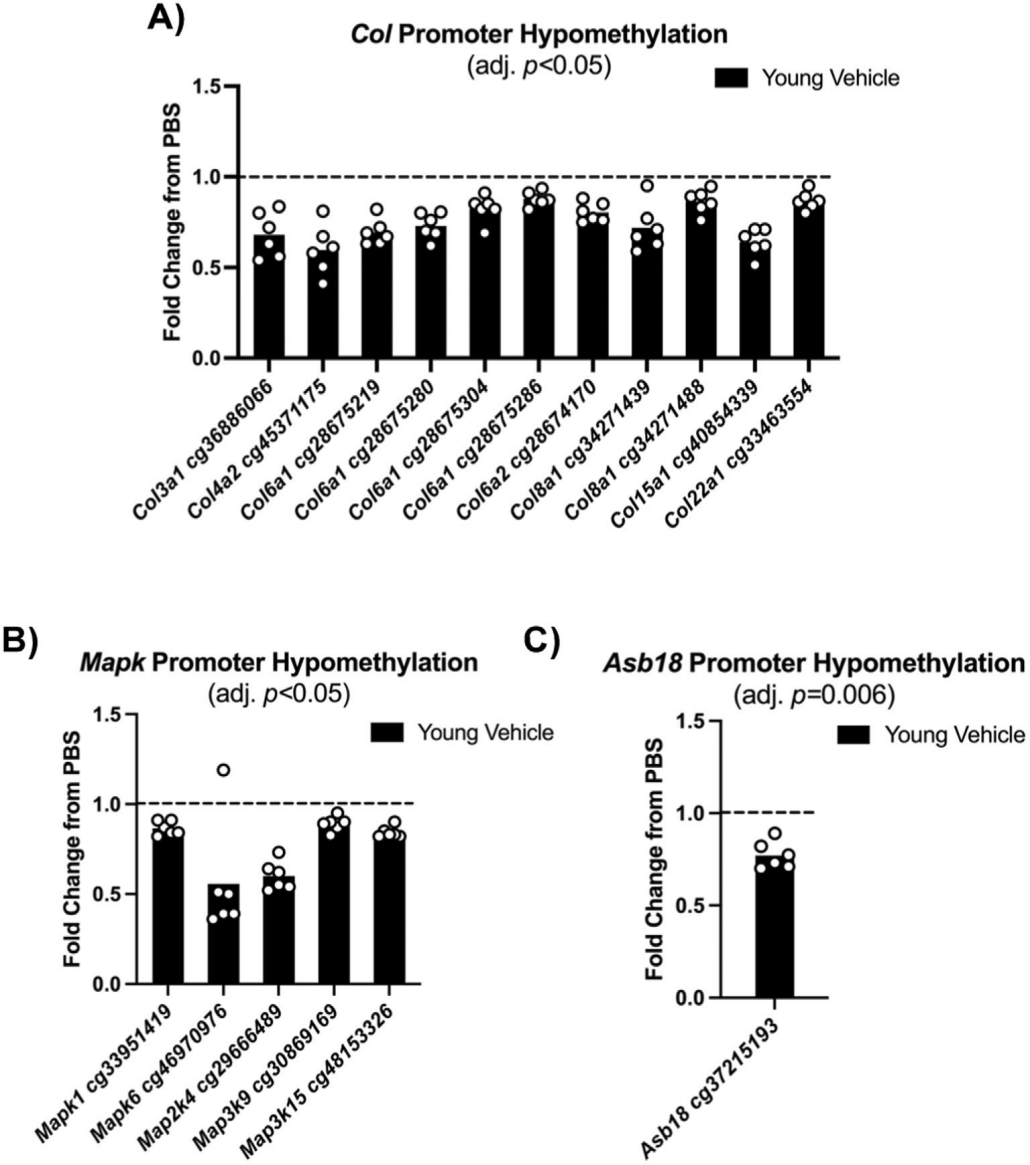
Methylation of conserved promoter CpGs is modified during muscle regeneration in young vehicle skeletal muscle. Promoter hypomethylation for young vehicle skeletal muscle for key gene families: *Col, Mapk, Asb18* (A–C). All promoter CpGs have an adj. *p <* 0.05, and individual fold change data points presented for young vehicle (*n* = 6) are relative to their respective control (PBS) contralateral limb.

In addition to characterizing the effects of senolytics on the molecular responses to muscle injury and repair, we sought to understand the effects of aging alone without the influence of senolytics. Relative to OV, there were fewer promoter region CpGs altered by degeneration than regeneration in YV (see Figure 2 and Table S2), but there were shared responses in young and aged muscle. Seven *Col* genes had hypomethylated promoters in YV muscle after regeneration: *Col3a1, Col4a2, Col6a1, Col6a2, Col8a1, Col15a1*, and *Col22a1* (adj. *p* value < 0.0; Figure 5A). Similar to aged muscle after regeneration, multiple hypomethylated promoter region CpGs were observed in YV for *Col6a1* and *Col8a1* (Figure 5A). In totality, however, the number of collagen genes affected by DNA methylation after regeneration was fewer in young versus aged muscle (see Figure 4A vs. Figure 5A) (Brack et al. 2007; Sousa‐Victor et al. 2022). These data are also generally consistent with collagen remodeling being a highly transcription‐driven process (Brightwell et al. 2022; Goetsch et al. 2003; Yan et al. 2003) that is dynamically regulated at the DNA methylation level (Zhang et al. 2017).

YV skeletal muscle had unique hypomethylation of promoter CpGs in members of the *Mapk* family of genes including *Mapk1, Mapk6, Map2k4, Map3k9*, and *Map3k15* (adj. *p* value < 0.05; Figure 5B). This pattern suggests potentially lasting epigenetic control of cell proliferation and differentiation after regeneration (Cargnello and Roux 2011; Guo et al. 2020). Promoter hypomethylation of *Asb18* specifically in YV (adj. *p* value < 0.05; Figure 5C) may have contributed to better skeletal muscle mass regulation or autophagy (Ehrlich et al. 2020; Gilbert et al. 2024; Gong et al. 2021) with regeneration relative to old, as this is what some ASB proteins in muscle have been linked to. This is speculative, however, since the specific role of *Asb18* in muscle is still undefined.

YV skeletal muscle did not feature promoter hypomethylation of *Hdac, Hox*, or *Wnt* genes after regeneration, which differs from the old group. However, after regeneration several *Hdac, Hox*, and *Wnt* genes were hypomethylated at exon, intron, intergenic downstream, 5′ UTR, and 3′ UTR CpG sites (Table S2). These data suggest differential regulation in young versus aged with regeneration, or could reflect accelerated recovery of methylation status in young mice.

### 3.5 Methylome‐Transcriptome Integration Predicts Epigenetic Regulation of Extracellular Matrix, Mapk Signaling Pathway, and Muscle Development Gene Expression in Young and Aged Muscle After Recovery From Injury

The relationship between gene expression and DNA methylation is not fully understood and at times seems paradoxical and counterintuitive (Bahar Halpern et al. 2014; López‐Moyado et al. 2019; Smith et al. 2020). For instance, promoter hypomethylation of a gene may or may not lead to elevated gene expression (Suzuki and Bird 2008; Thompson et al. 2010; Weber and Schübeler 2007). During skeletal muscle development, the relationship between the methylation of DNA promoter CpGs, as well as gene bodies to myogenic gene expression and regulation by methylation, is dependent on distance from the transcriptional start sites (TSS) (Yang, Fan, et al. 2021). Due to the complex and often unexplored/undefined role of DNA methylation on the regulation of gene expression, we employed a holistic computational approach called BETA that considers methylation patterns around a gene as well as proximity to the TSS to infer transcriptional regulation (Ismaeel et al. 2024). For this analysis, we focused on old versus young vehicle regeneration since the difference at the methylation level was more pronounced compared to the differences observed in OV versus OS.

**FIGURE 6:**
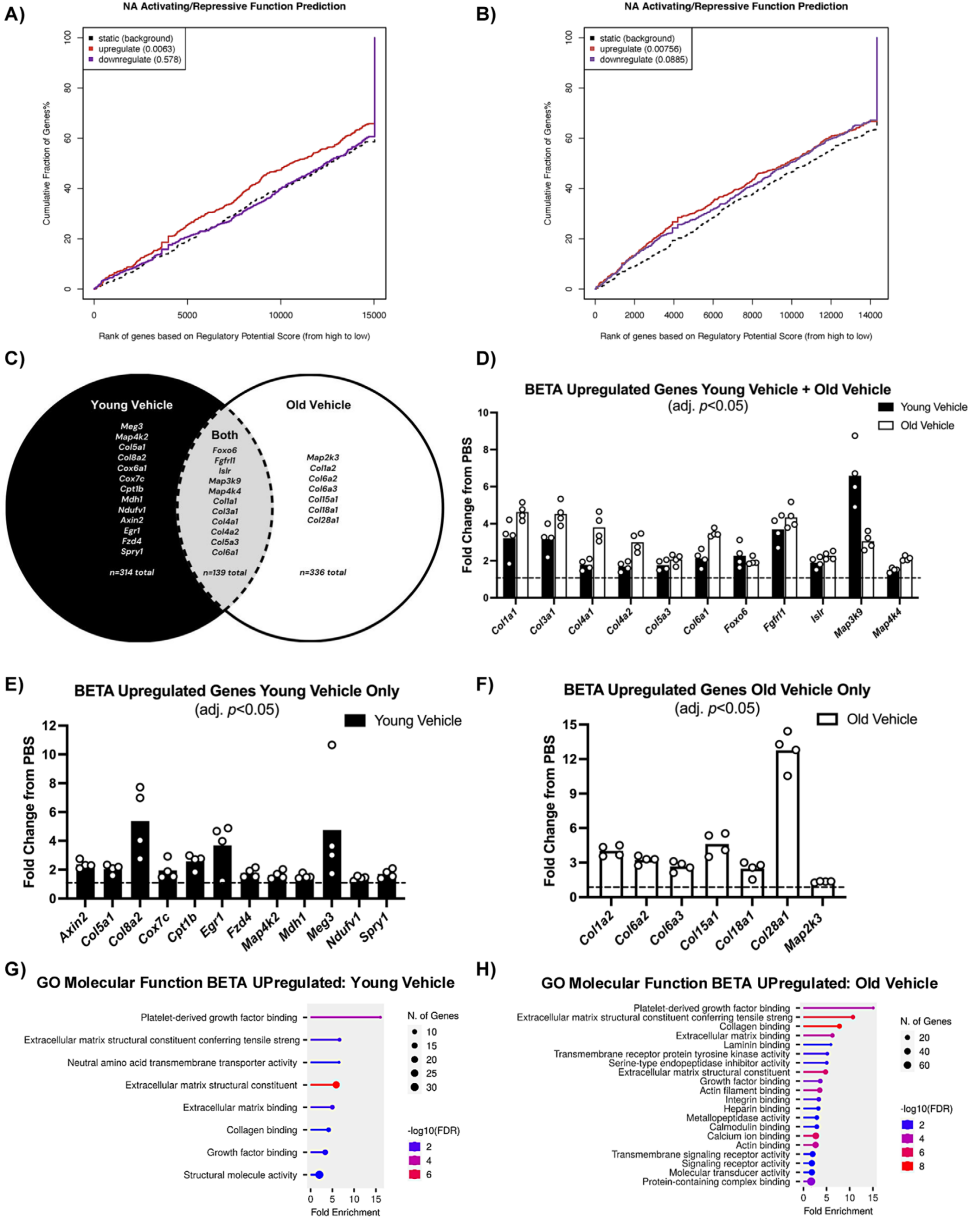
BETA integration of DNA methylome and transcriptome predicts epigenetic regulation of genes after muscle regeneration. (A) BETA analysis in young vehicle muscle of the relationship between CpG methylation status and upregulated (*p =* 0.006) and downregulated (*p =* 0.578) genes after muscle regeneration. (B) BETA analysis in old muscle of the relationship between CpG methylation status and upregulated (*p =* 0.008) and downregulated (*p =* 0.089) genes after muscle regeneration. (C) Venn diagram highlighting upregulated genes under epigenetic regulation determined by BETA for young vehicle, old vehicle, and both groups. (D) Key upregulated BETA genes after muscle regeneration in both young and old vehicle muscle related to extracellular matrix (ECM) integrity, tissue development, and cell signaling. (E) Key upregulated BETA genes after muscle regeneration in young vehicle muscle related to ECM integrity, cell signaling, and satellite cell health. (F) Key upregulated BETA genes after muscle regeneration in old vehicle muscle related to ECM integrity and cell signaling. (G) GO molecular function analysis of upregulated genes (*n* = 453) after regenerative in young muscle. (H) GO molecular function analysis of upregulated genes (*n* = 475) after regenerative in old muscle.

In YV, BETA analysis was significant for upregulated genes after muscle regeneration (*p* = 0.006, Figure 6A) but not for downregulated genes (*p* = 0.578, Figure 6A). In OV, BETA analysis was significant for upregulated genes after muscle regeneration (*p* = 0.007, Figure 6B) and was trending toward significance for downregulated genes (*p* = 0.089, Figure 6B). In YV and OV, DNA methylation status was predictive of upregulation in 453 and 475 genes, respectively, after regeneration (Table S3). Of the upregulated genes in YV and OV, there was overlapping expression of 139 genes between young and old muscle; thus, ~30% of the BETA upregulated genes were shared between YV and OV after regeneration (Figure 6C). OV had > 3 times more significant CpGs relative to YV after regeneration (~87,000 vs. ~24,000, respectively), but a similar number of differentially upregulated genes (~500 in YV and ~600 in OV, adj. *p* < 0.05). A comparable magnitude of agreement between DNA methylation changes and upregulated gene expression in OV and YV according to BETA suggests a more coordinated muscle epigenome‐transcriptome molecular response after regeneration in YV.

After regeneration, both young and old skeletal muscle had predicted methylation regulation of forkhead box O6 (*Foxo6*) transcript (adj. *p* value < 0.05; Figure 6D). *Foxo6* may have stimulated satellite differentiation and myotube formation since these processes are negatively affected in *Foxo6* loss of function models (Zhang et al. 2021). Unlike other members of the *Foxo* gene family that are associated with autophagy and delayed regeneration (e.g., *Foxo1* and *Foxo3a*), *Foxo6* is required for healthy regulation of cell metabolism and maintained myotube health and is implicated in protection against skeletal muscle atrophy by repressing genes associated with muscle wasting (Yamashita et al. 2016; Zhang et al. 2021). Fibroblast growth factor receptor‐like 1 (*Fgfrl1*) was upregulated and predicted to be controlled by methylation after muscle regeneration in both young and old (adj. *p* value < 0.05; Figure 6D). Slow muscle fiber development is regulated, in part, by *Fgfrl1* (Amann et al. 2014; Gerber et al. 2020; Giacomello et al. 2020). TA muscles have a low proportion of slow fibers in mouse; however, its upregulation may also play a role in the development and health of fast fiber types (e.g., IIX, IIB). A key gene implicated in satellite cell behavior that was predicted to be regulated by DNA methylation after regeneration in young and old was *Islr*. This gene is specifically associated with regulating *Wnt* signaling (Zhang, Zhang, et al. 2018) as well as asymmetric division in satellite cells (Liu et al. 2024). *Map3k9* and *Map4k4* were predicted to be regulated by DNA methylation in both YV and OV after regeneration; however, *Map4k2* was only regulated in young and *Map2k3* only in old muscle (Figure 6D–F; adj. *p* value < 0.05).

In young muscle, molecular function categories for BETA upregulated genes were largely related to platelet‐derived growth factor binding, serine transmembrane transporter activity and ECM structural constituents (Figure 6G). Similarly, in old muscle, molecular function categories for BETA upregulated genes were largely related to platelet‐derived growth factor binding and ECM structural constituents (Figure 6H). Several members of the *Col* gene families were upregulated in young and old skeletal muscle after regeneration and were predicted to be regulated by DNA methylation (adj. *p* value < 0.05; Figure 6F). More collagen genes were predicted to be regulated by DNA methylation in aged muscle after regeneration, however. *Col1a2*, *Col6a2*, *Col6a3*, *Col15a1*, *Col18a1*, and *Col28a1* were regulated in old (Figure 6H), whereas only *Col5a1* and *Col8a2* were predicted by BETA exclusively in young (Figure 6G).

### 3.6 Methylome‐Transcriptome Alterations Unique to Muscle of Young Mice After Regeneration

Upregulation of genes related to satellite cell function was predicted to be regulated by DNA methylation in both young and old muscle, but there were several noteworthy differences. Differential regulation of these certain genes may in part explain impaired or delayed regeneration that can occur in aged skeletal muscle. The top upregulated gene in YV, by adj. *p* value, was the long noncoding RNA *Meg3* (adj. *p* value = 0.00004; Figure 6E). This gene is heavily involved in muscle development and especially the proliferation of satellite cells (Dill et al. 2021; Yang, Liu, et al. 2021; Yao et al. 2023). *Meg3* was not regulated in OV at 35 days. Other upregulated genes specifically implicated in different aspects of satellite cell behavior that were only regulated by methylation in YV were *Axin2* (Figeac and Zammit 2015; Hulin et al. 2016; Huraskin et al. 2016), *Egr1* (Zhang, Tong, et al. 2018), *Fzd4* (Zhang, Yin, et al. 2023), and *Spry1* (Bigot et al. 2015; Chakkalakal et al. 2012; Shea et al. 2010). In young after regeneration, we also observed predicted methylation regulation of numerous metabolism and mitochondrial genes including *Cox6a1*, *Cox7c*, *Cpt1b*, *Mdh1*, *Ndufv1*, and others (Figure 6E; Table S3). This unique regulation may indicate metabolic rewiring in young mice after regeneration that is not reconstituted in aged mice.

## 4 Discussion

Regeneration after injury results in long‐term persistence of senescent cells in aged muscle (Dungan et al. 2022, 2021, 2023; Nolt et al. 2024). Removing senescent cells with the senolytic agent BI01 during muscle regeneration in aged mice facilitates recovery—this is demonstrated by larger muscle fibers, greater muscle mass and function, and more satellite cells relative to regenerated controls by 35 days (Nolt et al. 2024). Thus, DNAmAGE deceleration with senolytics observed here is consistent with the favorable phenotype findings we reported previously (Nolt et al. 2024). On balance, the effects of senolytics on DNAmAGE in muscle were fairly modest. This modest effect supports in vitro data suggesting that the methylation profile of senescent cells can be shared but is largely distinct from the *bona fide* CpGs that are altered by aging (Kabacik et al. 2018, 2022). In aged mice, regeneration had a profound impact on DNAmAGE deceleration regardless of senolytics. Changes to CpG methylation after muscle injury are likely essential to the redevelopment of skeletal muscle during regeneration. Deceleration of DNAmAGE in old mice tracks with these global methylation changes to CpG regions that likely promote tissue regeneration. There was a trend for decelerated DNAmAGE in regenerated skeletal muscle of young mice—specifically in the muscle intervention clock—but this was not statistically significant (*p* > 0.05). The global molecular shift after full histological and muscle mass recovery from injury is remarkable. There is widespread rewiring of the methylome and transcriptome in muscle tissue 35 days after injury regardless of age, but with a greater effect in aged. A recent investigation showed a memory of muscle regeneration that resulted in a unique and persistent post‐recovery methylome in muscle stem cells (Falick Michaeli et al. 2022), which aligns with earlier in vitro work (Sharples et al. 2016) and our current findings in whole tissue. In both young and aged mice, methylome and transcriptome integration revealed coordinated regulation of ECM and MAPK genes. Unique methylome and transcriptome changes in young versus aged mice further reinforce the cellular and molecular dysfunction that leads to impaired or delayed muscle healing in advanced age (Brunet et al. 2023; Sousa‐Victor et al. 2022).

Notable effects of senolytics during regeneration at the methylation level in muscle from aged mice were found in *Col*, *Hdac*, *Hox*, and *Wnt* genes. The difference in methylation of collagen genes with senolytics related to greater ECM remodeling, reinforcing a regulatory link between the methylome and the proteome in aged skeletal muscle (Chambers et al. 2024). Relatively more *Hdac* family genes with hypomethylation in promoter regions after regeneration with senolytics could be related to an improvement in satellite cell function (Brunet et al. 2023); this corroborates our previous observation with BI01 (Nolt et al. 2024). *Hdac* genes are essential to the epigenetic regulation of satellite cells and their ultimate ability to regenerate muscle (Massenet et al. 2021). Differential regulation of these genes after muscle tissue regeneration therefore seems intuitive. The epigenetic regulation of *Hox* genes is characteristic of aging (Haghani et al. 2023) and implicates developmental processes with advancing age (Horvath et al. 2023). *Hox* genes are also implicated in skeletal muscle adaptation to exercise in aged skeletal muscle (Murach, Dimet‐Wiley, et al. 2022; Turner et al. 2020). Perhaps epigenetic regulation of more *Hox* genes in the presence of senolytics during muscle regeneration could recapitulate some beneficial aspects of exercise—the most powerful countermeasure against muscle dysfunction with aging (Englund et al. 2021; Jones III et al. 2023; Murach, Dimet‐Wiley, et al. 2022; Smith et al. 2023; Voisin et al. 2024; Zhang, Englund, et al. 2022). Induction of the *Wnt* signaling pathway is implicated in tissue development and homeostasis, and *Wnt* genes are altered epigenetically in the muscle fiber with exercise (Wen et al. 2021). Activation of this pathway can induce satellite cell proliferation during skeletal muscle regeneration in adult mice (Liu et al. 2022; Otto et al. 2008). Methylation of *Wnt* pathway genes in skeletal muscle can also regulate satellite cell behavior (Zhang et al. 2019). It is worth mentioning that activation of the canonical *Wnt* signaling pathway leads to the fibrogenic conversion of aged satellite cells and can cause muscle fibrosis (Brack et al. 2007); however, functional satellite cells can be powerful mediators of proper ECM remodeling (Fry et al. 2017, 2014, 2015; Murach, Fry, et al. 2021; Murach et al. 2018; Murach, Peck, et al. 2021; Murach et al. 2020; Murphy et al. 2011), which is consistent with what we observe with senolytics here. Altogether, targeted methylation changes to regeneration‐relevant genes may represent a persistent signature of prior satellite cell identity and/or behavior and may explain why senolytic interventions improve healing in aged muscle (Jones III et al. 2024; Nolt et al. 2024).

By combining methylome and transcriptome data, we provide the first detailed information on the integrated molecular landscape of muscle tissue recovery after injury in young and old mice. Our analysis revealed that a similar number of genes had coordinated epigenome‐transcriptome regulation after regeneration in young and old (~450 genes; ~30% overlap), as well as shared regulation of ECM and MAPK genes regardless of age. There was also distinct regulation of collagen genes in young versus old after injury. Similar induction of *Foxo6*, *Fgfrl1*, and *Islr* in young and old reveals age‐independent epigenetic regulation in skeletal muscle that may promote tissue growth in response to muscle damage. Conversely, there was no lasting epigenetic signature in *Wnt* genes in young muscle in response to regeneration. The methylation patterns and expression of several satellite cell‐related genes (e.g., *Axin2*, *Egr1*, *Fzd4*, *Meg3*, *Spry1*) call attention to divergent epigenetic responses in the regeneration of young and old muscle. Some possibilities for the persistent regulation of these genes after regeneration are that it could: (1) prime the response of satellite cells to subsequent repeated injury (Falick Michaeli et al. 2022; Morroni et al. 2023), (2) be related to the molecular landscape of other cells in muscle after regeneration such as FAPs or macrophages, or (3) represent an epigenetic “memory” of satellite cell behavior during regeneration that persists in the myonuclei of the regenerated muscle fiber (Murach, Dungan, et al. 2022). These age‐dependent differences may have implications for future studies of skeletal muscle therapeutic approaches to accelerate muscle recovery.

The consequences of decelerated DNAmAGE and persistent epigenetic differences after regeneration in aged skeletal muscle tissue deserve further exploration. Injury‐experienced satellite cells may have improved performance with repeated injury (Falick Michaeli et al. 2022; Morroni et al. 2023); [Correction added on 13th May 2025 after first online publication: The reference citations “Gerber et al. 2020” and “Giacomello et al. 2020” have been replaced with “Falick Michaeli et al. 2022” and “Morroni et al. 2023.”] however, it is uncertain whether an injured then regenerated skeletal muscle has a practical adaptive advantage over an uninjured muscle regardless of lower DNAmAGE. For instance, following regeneration, some evidence suggests that muscle loses the ability to respond to hypertrophic mechanical overload in young rodents (Kawano et al. 2017). This observation points to impaired plasticity and a more influential role for resident myonuclei—as opposed to satellite cells and their progeny—in muscle adaptation. Perhaps persistent differences in the myonuclear DNA methylome and transcriptome after regeneration explain this lack of plasticity, which coincides with a tendency toward accelerated DNAmAGE in muscle tissue of young mice observed here. Regardless, the beneficial effects of senolytics for muscle healing extend to the epigenetic level and could facilitate future muscle adaptability in aged muscle. We cannot unravel the potential cellular impact of senolytics independent from their role in removing senescent cells in the current investigation, but our data provide in vivo information that helps inform the relationship between senescent cells and methylation aging. We also provide a resource for understanding the epigenetic regulation of gene expression after regeneration in young versus aged mice, which may help identify new targets to improve muscle healing potential throughout the lifespan.

### 4.1 Study Limitations

Senolytic (BI01) administration leads to an increased proportion of macrophage cell types in skeletal muscle during regeneration (Nolt et al. 2024). There remain questions whether the benefits of senolytics are the result of decreased senescent cells or accumulation of other cell types. Future investigations of individual cell type methylation patterns after senolytic administration, as well as myonuclei, would help define their actions and roles in response to skeletal muscle injury and regeneration. Additionally, full recovery from injury between young and old muscle may vary and should be further studied. It is unclear as of now if DNAmAGE from old, injured muscle remains lower for perpetuity relative to the uninjured limb after 35 days of regeneration. Furthermore, the effects of serial BaCl_2_ injury on DNAmAGE and CpG methylation warrant investigation to determine how skeletal muscle responds to repeated insults and if an “epigenetic memory” of injury is retained. Such a “memory”—specifically in satellite cells—could reduce the deleterious effects of each subsequent injury. Lastly, implementation of different injury models (i.e., freeze, impact, volumetric muscle loss, etc.) should be considered to test the efficacy of senolytics during regeneration, as there can be differences in how recovery from these injuries ensues (Hardy et al. 2016).

## Author Contributions

C.M.D. and K.A.M. conceived the study. T.L.C., J.W., P.J.K., F.M., Z.B.M., R.T.B., J.G., M.M., S.H., and Y.W. performed experiments and/or analysis and/or data interpretation. T.L.C. and K.A.M. wrote the manuscript draft with input from C.M.D., K.A.M., and C.M.D. provided resources and oversight. All authors reviewed and approved the manuscript.

## Ethics Statement

All animal procedures were approved by the Institutional Animal Care and Use Committee of the University of Kentucky.

## Consent

The authors have nothing to report.

## Conflicts of Interest

Y.W. is the founder of MyoAnalytics LLC. The Regents of the University of California are the sole owners of patent applications directed at the Horvath mammalian methylation array and various epigenetic clocks for which Steve Horvath is a named inventor. S.H. and R.T.B. are co‐founders of the non‐profit Epigenetic Clock Development Foundation that licenses these patents and distributes the mammalian array. S.H. is a principal investigator at Altos Labs. The remaining authors have no other competing interests to declare.

## Supporting information


**Table S1.** Horvath mammalian mDNAge individual clock ages (yrs) of old senolytic, old vehicle and young vehicle groups with PBS and BaCl2 injections of the tibalias anterior. Presented are the individual sample IDs and results for each Horvath mDNAge clock of interest.
**Table S2.** Methylation at CpG sites in the YV, OV, and OS groups with muscle regeneration (PBS and BaCl_2_). Individual CpG methylation for young and old mice are included for reference. Results describe the CpG ID, gene symbol, CpG type, *p* value and adj. *p* values for comparison of BaCl_2_ to PBS, and group averages for comparison. *NA = gene symbol not specified.
**Table S3.** BETA integration of the skeletal muscle methylome regions upstream of the transcriptional start site and transcriptome during muscle regeneration in young and old mice. Chromosome number, start and end sites from the array, RefSeq ID, rank product, and gene symbol are all presented. The overlap of the BETA upregulated genes are also presented for young and old mice with muscle regeneration.

## Data Availability

The data that support the findings of this study are available in the Supporting Information of this article. RNA sequencing data have been deposited to NCBI Gene Expression Omnibus GEO under accession number GSE294565 (Nolt et al. 2024). Methylation array data are deposited in GEO under accession number GSE290909.
